# OLDER ADULTS’ ONLINE INFORMATION SEEKING FOR ALZHEIMER'S DISEASE

**DOI:** 10.1093/geroni/igac059.1879

**Published:** 2022-12-20

**Authors:** Matthew Picchiello, Payton Rule, Brian Carpenter

**Affiliations:** Washington University in St. Louis, St. Louis, Missouri, United States; Washington University in St. Louis, St. Louis, Missouri, United States; Washington University in St. Louis, St. Louis, Missouri, United States

## Abstract

Many older adults use the internet to gather health information. Yet past research shows that older adults display less understanding of internet concepts and score lower on information-retrieving tasks than younger adults, making them more vulnerable to internet misinformation. To address this, a variety of guidelines (e.g., HONCode) have been developed to help consumers identify credible sources. This is especially relevant for Alzheimer disease (AD), as research has shown that online AD information is mixed in its quality. The purpose of this study was to examine how older adults gather online AD information and evaluate their ability to find credible sources to improve knowledge. We recruited 83 older adults (Mage = 70.0, 60.2% female; 85.5% White) who completed the Alzheimer Disease Knowledge Scale (ADKS; Carpenter et al., 2009) before and after engaging in six AD-related search tasks (e.g., identify age of onset, risk factors, treatment, etc.). Older adults utilized a variety of search strategies, visiting both reputable (i.e., not-for-profit or university-based) and less scrutinized (i.e., commercially sponsored) websites, and a vast majority (95.0%) were unaware of HONCode guidelines for evaluating website quality. While most participants could locate some AD-related information (97.6% identified risk factors), other information was more elusive (29.3% could not identify age of onset). Despite this, participants showed improved ADKS scores, t(74) = 5.55, p < 0.001. Our research shows that many older adults can gather some accurate online information about AD but may benefit from additional resources on using effective search strategies and identifying credible sources.

